# Ripe Tomato Detection Algorithm Based on Improved YOLOv9

**DOI:** 10.3390/plants13223253

**Published:** 2024-11-20

**Authors:** Yan Wang, Qianjie Rong, Chunhua Hu

**Affiliations:** College of Information Science and Technology & Artificial Intelligence, Nanjing Forestry University, Nanjing 210037, China; 2210610419wy@njfu.edu.cn (Y.W.); 18851728716@163.com (Q.R.)

**Keywords:** ripe tomatoes, YOLOv9, fruit detection, HGBlock, SPD-ADown

## Abstract

Recognizing ripe tomatoes is a crucial aspect of tomato picking. To ensure the accuracy of inspection results, You Only Look Once version 9 (YOLOv9) has been explored as a fruit detection algorithm. To tackle the challenge of identifying tomatoes and the low accuracy of small object detection in complex environments, we propose a ripe tomato recognition algorithm based on an enhanced YOLOv9-C model. After collecting tomato data, we used Mosaic for data augmentation, which improved model robustness and enriched experimental data. Improvements were made to the feature extraction and down-sampling modules, integrating HGBlock and SPD-ADown modules into the YOLOv9 model. These measures resulted in high detection performance with precision and recall rates of 97.2% and 92.3% in horizontal and vertical experimental comparisons, respectively. The module-integrated model improved accuracy and recall by 1.3% and 1.1%, respectively, and also reduced inference time by 1 ms compared to the original model. The inference time of this model was 14.7 ms, which is 16 ms better than the RetinaNet model. This model was tested accurately with mAP@0.5 (%) up to 98%, which is 9.6% higher than RetinaNet. Its increased speed and accuracy make it more suitable for practical applications. Overall, this model provides a reliable technique for recognizing ripe tomatoes during the picking process.

## 1. Introduction

Tomatoes, with their vibrant colors, are rich in numerous nutrients [1]. In 2020, the global tomato cultivation area reached an impressive 5.0305 million hm^2^ [2]. Currently, there are two types of ripe tomato detection: manual detection and machine learning. However, traditional manual harvesting is inefficient and costly, which makes it difficult to meet large-scale cultivation demands. Manual picking results in overripe tomatoes not being picked and potentially wasted due to the picking motion. Automating tomato detection can improve these problems and increase agricultural efficiency.

With the development of deep learning, target detection methods are being applied to fruit detection. They have significantly advanced digital agriculture [3], and computer vision applications powered by deep learning are expanding rapidly within the agricultural sector [4]. Several classical detection algorithms are used for fruit detection, with notable examples including You Only Look Once (YOLO), the region convolutional neural network (R-CNN), the fast region convolutional neural network (Fast-RCNN) [5], and the faster region convolution neural network (Faster-RCNN) [6]. Convolutional neural network-based target detection algorithms are mainly categorized into two-stage and single-stage phase algorithms. The two-stage algorithms are relatively slow as the whole algorithm is processed in two stages. For example, Yu et al. [7] applied the Mask-RCNN algorithm for strawberry picking and achieved an accuracy rate of 95.78%, but the detection speed was slow due to the large amount of computation. Lochan et al. [8] used an enhanced Fast-RCNN model that not only improved the efficiency of the training process and classification by a factor of 200 but also provided 96–97% accuracy. Gao et al. [9] achieved high accuracy in recognizing obscured apples using Faster R-CNN with 0.879 mAP and an improved detection time of 0.241 s, but the detection time was still long. Recently, single-stage object detection models, such as the YOLO series, Single Shot MultiBox Detector (SSD) [10], and RetinaNet [11], have addressed the issue of slow detection while maintaining reasonable accuracy. Ali et al. [12] employed SSD for fruit detection and the confidence value of the model’s performance ranged from 75% to 99% under adequate illumination conditions. On the other hand, the model’s performance degraded under sub-optimal illumination conditions. Conversely, Agarwal et al. [13] used SSD to detect mangoes on canopies and achieved 92.43% mAP after the color transformation of the dataset. Wang et al. [14] used YOLOv3, which achieved a mAP and detection time of 96.41% and 20.28 ms, respectively, earlier than Agarwal.

With advancements in YOLO, detection models have undergone significant optimization. The YOLO algorithm was originally faster than other target detection algorithms [15]. You Only Look Once version 9 (YOLOv9), with its iterative improvements, has notably enhances accuracy and detection speed. It can maintain high detection rates for a wider range of targets at a wide range of scales while maintaining high speeds. Lawal et al. [16] applied a label-what-you-see approach, dense architecture integration, spatial pyramid pooling, and Mish function activation to modify YOLOv3 for tomato detection, increasing the mAP to 98.4%. Chen et al. [17] incorporated K-Means clustering into DIoU NMS for prune detection using YOLOv4 with a recall of 96.03%, but the accuracy for complex scenes must be improved. Wang et al. [18] proposed an automatic tomato detection method based on the YOLOv8 model with a 1.5% increase in mAP over the pre-improvement period, which reduces the model size but decreases the speed when detecting complex data. As machine learning continues to develop, it becomes increasingly effective at addressing real-world problems. However, the accuracy and speed of object detection for small objects and complex environments must be further improved. YOLOv9 has four models with different parameters and YOLOv9-C is an open-source model. YOLOv9-C is able to learn finer features in the input image for small targets.

Even though YOLO has been updated with multiple versions, it still faces some challenges, including (1) the detection accuracy of objects with different shapes, sizes, brightness, and colors must be improved; (2) small objects are not detected with high accuracy; (3) the complexity of the algorithm is high and requires high GPU performance. To address these issues, this paper proposes to detect ripe tomatoes based on an improved YOLOv9-C model that fuses HGBlock and SPD-ADown to improve detection speed and accuracy.

The optimal improvement method is derived from comparing other module replacement methods. The main contributions of this paper are as follows: (1) The HGBlock module from the RT-DETR network was used to replace the RepNCSPELAN4 feature extraction module in YOLOv9. This module reduced both computational cost and algorithm size while improving detection speed to 14.7 ms, thereby solving the problem of complex algorithms and high equipment requirements. (2) The SPD-ADown model is integrated into YOLOv9, enhancing model robustness, detection accuracy, and suitability for tomato picking with mAP@0.5 (%) up to 98%, thereby addressing YOLOv9’s limitations in small object recognition. (3) A series of experiments validated the effectiveness of the proposed algorithm.

## 2. Materials

### 2.1. Collection

In this paper, tomato image data were collected from the greenhouse at the Nanjing Institute of Vegetable and Flower Science using both a consumer-grade digital camera and an Intel RealSense camera. The specific parameters of the Intel Realsense D435 camera (Intel Corporation, Zhongshan, China) are shown in Table 1.

**Figure 1:**
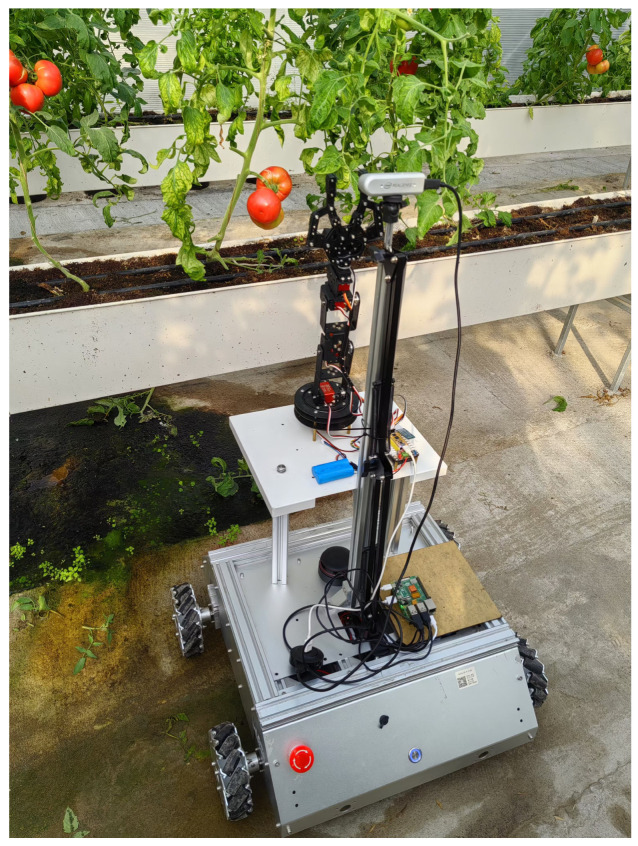
Tomato data picking scene.

**Figure 2:**
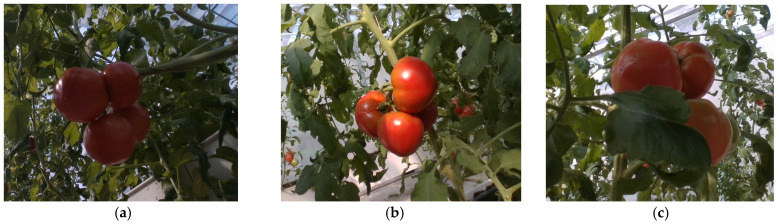
Partial dataset of tomato sampling in different environments: (**a**) backlight; (**b**) light; (**c**) tomatoes covered by leaves.

Figure 1 illustrates the scene during data collection. Photographs were taken from 9:00 a.m. to 11:00 a.m. and from 3:00 p.m. to 5:00 p.m. during October 2023 to ensure a greater variety of light and tomato ripening conditions. A total of 698 RGB images and corresponding depth images of tomato fruits were gathered. To enhance dataset diversity and enable the model to learn as much as possible about the fruit’s features, variations in lighting conditions were considered during image acquisition, including backlighting, direct lighting, and different occlusion scenarios. In Figure 2, an example of a partial dataset is shown with an image resolution of 640 × 480. This resolution is sufficient to meet the needs of the YOLOv9 model while saving the space it occupies.

In this study, we used Labelimg to annotate the image dataset. Labelimg is a visual image annotation tool written in Python and built with a QT interface [19]. It supports formats such as PASCAL VOC and YOLO, making it suitable for target detection networks such as Faster-RCNN, SSD, and YOLO. The annotations in this paper are in YOLO format, which records category and bounding box information. In order to ensure quality annotations, labels were annotated by a single person using a uniform standard avoiding discrepancies. When encountering small objects or occlusions, only the part that appears is labeled, with the same Group ID for the different parts.

### 2.2. Data Augmentation

**Figure 3:**
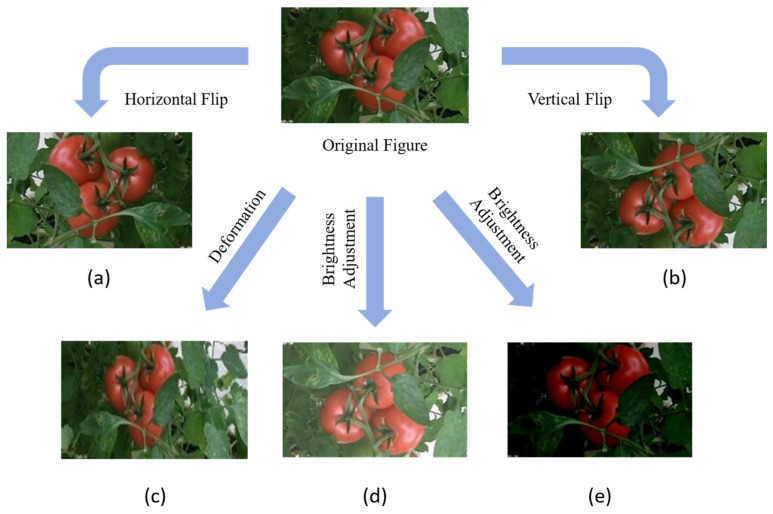
Data augmentation: (**a**) horizontal Flip; (**b**) vertical flip; (**c**) deformation; (**d**) brightness adjustment; (**e**) brightness adjustment.

To enhance image features, prevent overfitting, and improve model robustness, this study employed the Augmentor tool for data augmentation prior to network training. Augmentor is a Python library designed for image augmentation in computer vision projects. Tomatoes grown together are susceptible to light shading, leaf shading, and tilted fruit growth during detection. Augmentor offers a range of user-friendly methods and tools to perform various augmentation operations on images. In this study, the dataset was expanded through techniques such as horizontal and vertical flipping, deformation, and brightness adjustment. This data enhancement increased the dataset to 1100 images. Figure 3 illustrates an example of the original image alongside the tomato image after data augmentation.

This paper also uses the Mosaic data augmentation method, which was first proposed in a YOLOv4 paper. The core idea is to randomly select four images from the overall dataset, crop them according to randomly generated crosshairs, and stitch the cropped parts together [20]. Additionally, the target box on each original image is limited by the crosshair crop.

**Figure 4:**
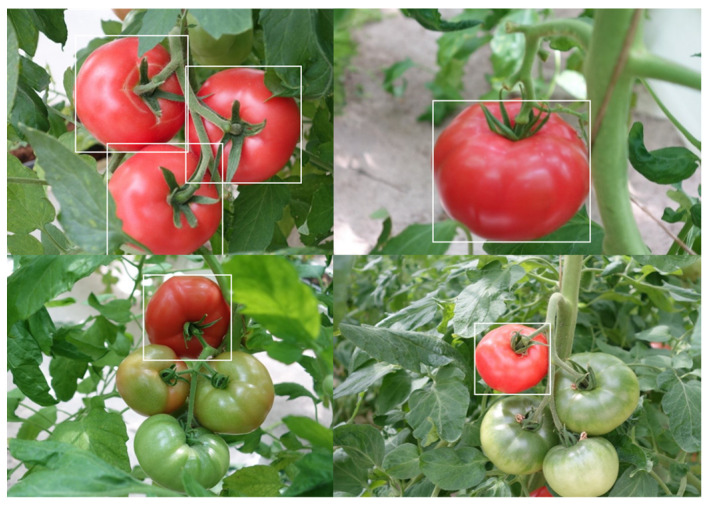
An example of Mosaic data augmentation.

Mosaic data augmentation involves inputting four images simultaneously during training, effectively increasing the sample size for each session while randomly resizing large samples into smaller ones. This approach boosts the number of small targets and enhances the model’s recognition capabilities. Mosaic data augmentation generates more training samples by combining multiple images, which improves the model’s generalization ability. It helps the model better understand the relationships between different objects. It also adapts to variations in target size, pose, and background, thereby enhancing target detection performance. Mosaic scales the image before stitching to ensure that small targets do not lose important features. After stitching, Mosaic adjusts the bounding box coordinates to ensure that the labeling information is correct. Figure 4 illustrates an example of Mosaic data augmentation with white boxes representing labeled real values. By combining traditional data augmentation techniques with the Mosaic algorithm, the training set was expanded to 1800 images, creating a comprehensive tomato dataset suitable for this paper’s algorithm, with the dataset divided into 80% for training and 20% for testing.

## 3. Methods

### 3.1. Framework of YOLOv9

Among the various target detection algorithms, the YOLO framework is notable for its exceptional balance of speed and accuracy. Introduced in 2016 by Joseph Redmon and colleagues, the YOLO algorithm pioneered single-stage and real-time target detection. It achieves target localization and classification through regression, which substantially reduces computational effort and significantly enhances detection speed. However, it tends to perform less effectively at detecting small or overlapping targets.

YOLOv9 offers several significant improvements over previous YOLO models [21]. While existing deep learning algorithms primarily focus on designing suitable objective functions and architectures to enhance prediction accuracy, they often overlook the substantial information loss that occurs during layer-by-layer feature extraction and spatial transformation. To tackle this issue of data loss in deep learning networks, the YOLOv9 team offers two key contributions:

(1) Programmable Gradient Information (PGI) was introduced as a novel auxiliary supervision framework that generates reliable gradient information for updating network weights during training. PGI addresses the challenges posed by deep networks by supporting reversible branching and ensuring complete input information for computing the objective function [22];
(2) A new gradient-based path planning network architecture, GELAN, was designed. GELAN improves parameter efficiency using only standard convolutional operators.

By integrating the proposed PGI and GELAN, YOLOv9 surpasses existing real-time object detectors for target detection in MS COCO datasets. We developed an enhanced tomato fruit detection model based on YOLOv9 to establish a foundation for tomato localization.

**Figure 5:**
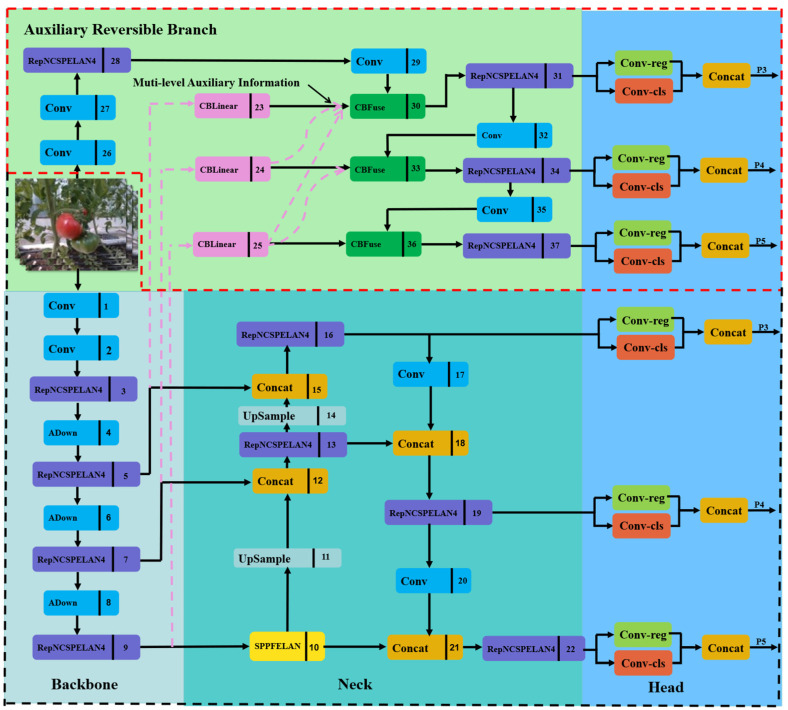
The framework of the YOLOv9 network.

YOLOv9, introduced by the YOLOv7 team in 2024, is the latest object detection network. It consists of five models with varying parameter counts: YOLOv9-T, YOLOv9-S, YOLOv9-M, YOLOv9-C, and YOLOv9-E, listed in ascending order of parameter count. YOLOv9-C has advantages in small target detection and is suitable for detecting ripe tomatoes. Therefore, we selected YOLOv9-C as the base framework; its specific structure is illustrated in Figure 5.

YOLOv9 incorporates PGI structure, which comprises three main components: the main branch, the Auxiliary Reversible Branch, and Multi-level Auxiliary Information. In Figure 5, the main branch is highlighted within a black box, while the Auxiliary Reversible Branch and Multi-level Auxiliary Information are enclosed in a red box. During training, the Auxiliary Reversible Branch and Multi-level Auxiliary Information are crucial. As the network deepens, an information bottleneck may arise, leading to unreliable gradients from the loss function. To minimize the loss of information on the main branch, the integrity of the information is maintained through the reversible function of the Auxiliary Reversible Branch. Additionally, Multi-level Auxiliary Information addresses the error accumulation problem in deep supervision mechanisms and enhances model learning by incorporating various levels of auxiliary information. During inference, only the main branch is utilized, so there are no extra inference costs.

**Figure 6:**
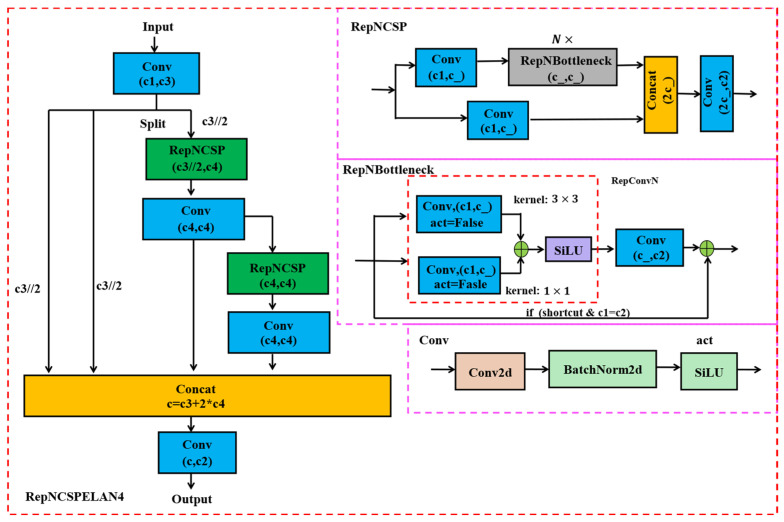
The structure of RepNCSPELANA4.

As depicted in Figure 5, YOLOv9 enhances the efficiency of information integration and propagation during model training by incorporating a new lightweight network architecture, GELAN, represented as RepNCSPELAN4. GELAN combines CSPNet and ELAN to efficiently aggregate network information, minimize information loss during propagation, and improve inter-layer information interaction. Its reduced parameters and computational complexity make it especially suitable for environments with limited computational resources. The structure of RepNCSPELANA4 is illustrated in Figure 6.

**Figure 7:**
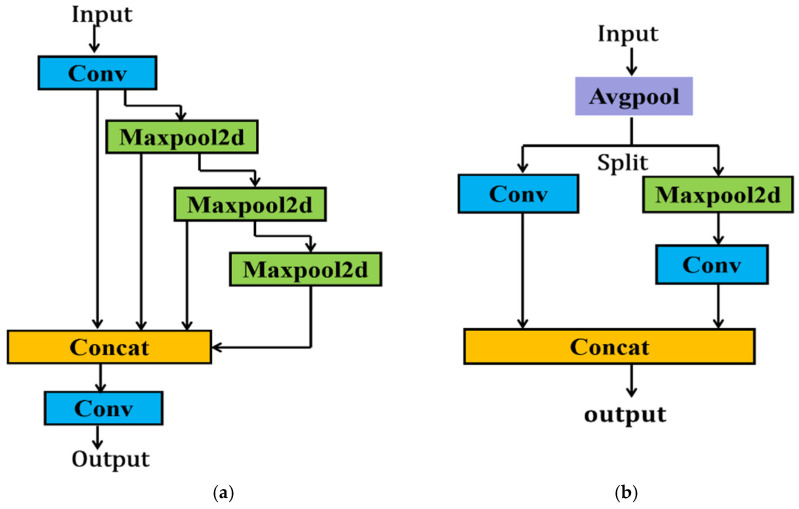
Modules in YOLOv9: (**a**) SPEELAN module; (**b**) ADown module.

YOLOv9 incorporates CBLinear and CBfuse modules from the DynamicDet project, introducing auxiliary reversible branches. Additionally, the SPEELAN module is introduced, which combines SPP and ELAN (Efficient Local Aggregation Network) to enhance target detection by leveraging the strengths of both. Figure 7a illustrates the structure of the SPEELAN module. The ADown module, designed for downsampling between different layers of the feature map, is depicted in Figure 7b.

### 3.2. Improvement of Feature Extraction Module

**Figure 8:**
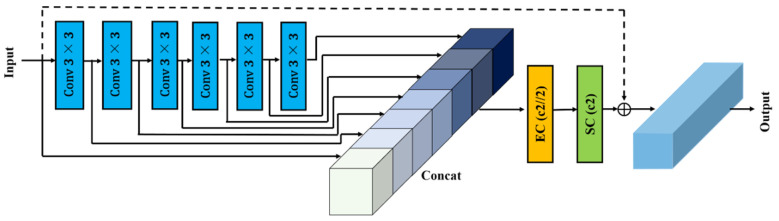
Structure of the HGBlock module.

The RepNCSPELAN4 module was used in the feature extraction component of the original YOLOv9 network. In this study, the HGBlock module from the RT-DETR network replaced the RepNCSPELAN4 feature extraction module [23]. For GPU devices, RT-DETR authors analyzed and summarized current GPU-optimized networks, determining that maximizing the use of “3 × 3” standard convolutions (which offer the highest computational density) is beneficial for GPU inference while maintaining high accuracy. HGBlock integrates numerous “3 × 3” convolutional modules along with channel compression (EC) and decompression (SC) modules, as illustrated in Figure 8.

In the figure, Conv “3 × 3” denotes a module with a convolutional layer, a BatchNorm2d layer, and an activation function layer, all at a size of “3 × 3”. The primary function of the channel compression module is to reduce the number of channels to half of the target output channels, while the decompression module adjusts the number of channels to meet the target output channels. This paper analyzes the performance of these modules through ablation experiments.

### 3.3. Improvement of Down-Sampling Module

**Figure 9:**
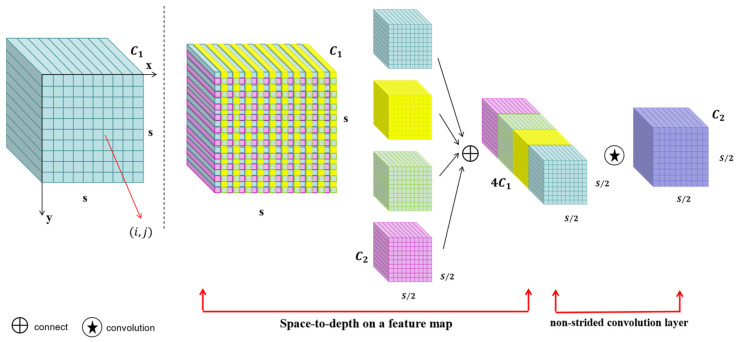
SPD-conv module.

Ding et al. [24] integrated SPD-Conv into the YOLO algorithm and improved its accuracy by 4%. Inspired by this experiment, we integrated the SPD-Conv module into YOLOv9 to improve its detection accuracy. SPD-conv consists of a space-to-depth (SPD) layer and a non-strided convolution layer [25], as shown in Figure 9. SPD-conv slices an intermediate feature mapping *X* of arbitrary size *S* × *S* × *C*_1_, as represented by the following formulae:

(1) $$f_{0,0}=X\left[0:S:scale,\;0:S:scale\right],\;f_{1,0}=X\left[1:S:scale,\;0:S:scale\right],\ldots ,\;f_{scale-1,0}=X\left[scale-1:S:scale,\;0:S:scale\right];$$

(2) $$f_{0,1}=X\left[0:S:scale,\;1:S:scale\right],\;f_{1,1}=X\left[1:S:scale,\;1:S:scale\right],\ldots ,\;f_{scale-1,1}=X\left[scale-1:S:scale,\;1:S:scale\right];$$

⋮

(3) $$f_{0,scale-1}=X\left[0:S:scale,\;scale-1:S:scale\right],\;f_{1,0},\ldots ,\;f_{scale-1,\;scale-1}=X\left[scale-1:S:scale,\;scale-1:S:scale\right];$$

As seen in Figure 9, when scale=2, four sub-feature mappings $f_{0,0},\;f_{0,1}f_{1,0}f_{1,1}$ can be obtained with shape size ($S/2,S/2,C_{1}$). These sub-feature mappings are then connected along the channel dimensions to obtain a feature mapping $X^{\prime }$. After the SPD feature transformation layer, a non-strided convolution layer is added, eventually changing the feature layer from $X\;(S,S,C_{1})$ to $X^{\prime \prime }\left(S/scale,S/scale,C_{2}\right)$.

In traditional CNN architectures, if the step-length convolution and pooling layers are applied directly, the spatial resolution of the image decreases as the network level deepens, resulting in a loss of detailed information about small objects and making it difficult for the network to accurately recognize these small objects [26]. This combined use of SPD layers and non-step-length convolutional layers allows the convolutional neural network to better handle challenges with small objects and low-resolution images, improving the model’s performance and robustness in complex scenes.

**Figure 10:**
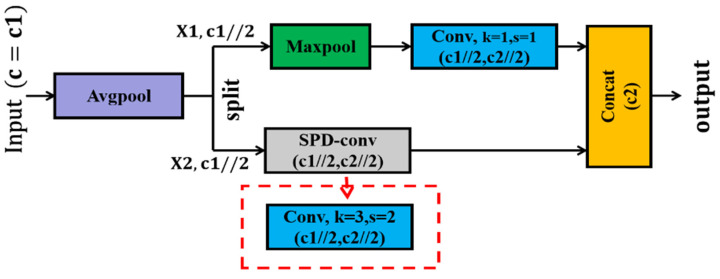
SPD-ADown module.

The ADown module consists of an average pooling layer, a maximum pooling layer, and a convolutional layer. In this study, we improved the ADown module in the original YOLOv9 based on the module. As shown in Figure 10, the SPD-conv module is used to replace the convolutional layer with the original kernel size of 3 and step size of 2 [27].

**Figure 11:**
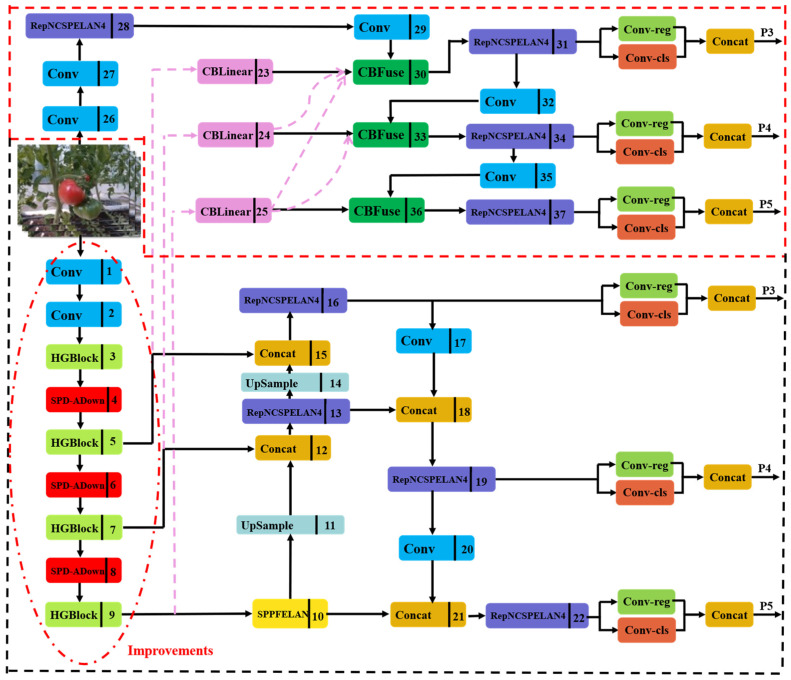
Improved YOLOv9 model.

Finally, we integrated the HGBlock and SPD-ADown modules into YOLOv9 model. The overall structure of the improved model is shown in Figure 11. The red box shows the location where each module is added in this paper.

### 3.4. Evaluating Indicator

In order to objectively measure the detection performance of the network model on the target, metrics such as Recall, Precision, AP value, detection speed, number of total model parameters, and model size are used to evaluate the model [28]. Among them, Recall, Precision, and mAP are calculated as follows:

(4) $$Recall=\frac{TP}{TP+FN}$$

(5) $$Precision=\frac{TP}{TP+FP}$$

(6) $$AP=\int_{0}^{1}P\cdot RdR$$

(7) $$mAP=\frac{\sum_{i=1}^{N}AP_{i}}{N}$$

TP denotes the number of positive samples predicted as positive classes by the model. FN denotes the number of positive samples predicted by the model to be in the negative category. FP represents negative samples that are predicted as positive classes by the model. N represents the number of classes in the sample. Precision represents the proportion of positive samples predicted as truly positive samples. The higher the precision, the higher the proportion of positive samples predicted by the model as truly positive samples, and the lower the model’s misdetection rate is indicated [29]. Recall reflects the model’s ability to find all true positive samples and is often used to assess underdetection. The average precision AP is obtained by calculating the area enclosed by the model precision and recall curves with the axes. P and R in Equation (6) are shorthand for precision and recall. mAP represents the average of the AP calculated over all categories and is used to measure how accurate the trained model is overall across categories [30]. mAP@0.5 and mAP@0.5:0.95 represent the average mAP value when the IoU threshold is set to 0.5 and the thresholds range from 0.5 to 0.95.

In order to understand the contribution of each component of the model, we designed a series of ablation experiments. In the experiments, to ensure the reliability of the results, all the models were trained with a unified hyperparameter configuration. The specific parameters are shown in Table 2. The input size of the image was set to the standard size of 640 × 640. The number of categories was set to 1, the total number of training rounds was 200, and the number of samples was set to 4 for each training. The optimizer for training was SGD optimizer, the initial learning rate was 0.01, and the momentum was set to 0.937. These parameters were selected manually based on previous experience. The selected parameters were also fine-tuned with an optimizer to find the best combination to further improve their accuracy.

## 4. Experimental Results and Analysis

### 4.1. Ablation Experiment

The original YOLOv9-C was used as the benchmark model in this study. In order to verify the validity of different modules, we used mAP@0.5:0.95, mAP@0.5, Precision, and Recall as evaluation metrics of the model performance on the tomato dataset of this paper [31]. The results of the ablation experiments are shown in Table 3. As seen in Table 3, mAP@0.5 in the metrics of the model improves by 4.8% after adding the SPD-ADown module, while the Recall of the model improves by 1.2% after integrating HGBlock. The model enhancement is even more significant after adding both SPD-ADown and HGBlock modules, with 5% and 1.1% improvement in mAP@0.5 and mAP@0.5:0.95 over the baseline model, respectively. Precision and Recall also improved by 1.3% and 1.1%, respectively.

**Figure 12:**
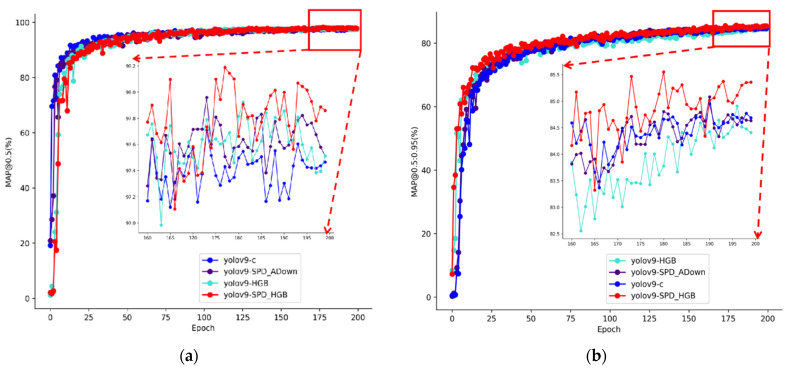
The performance of each model under different metrics: (**a**) mAP@0.5; (**b**) mAP@0.5:0.95.

In order to further verify improvements in the detection ability of the improved algorithm, the change in evaluation indexes between the enhanced algorithm and the benchmark algorithm during the training process was analyzed through experiments. As shown in Figure 12, the trend change curves of various data in the model test set during its training process were recorded by mAP@0.5 and mAP@0.5:0.95. A total of 200 rounds of testing were conducted. From the figure, it can be seen that all models converge and the improved model has higher detection accuracy.

Meanwhile, this paper evaluates each improved model based on inference time, Params, and FLOPs. The results are shown in Table 4. Params refer to the model’s number of parameters, which is often used to measure the size and complexity of the model. FLOPs (Floating Point Operations) are a measure of the computational complexity of computer algorithms [32]. It refers to the number of floating point operations performed during the arithmetic process. Combining the individual mAP, precision, and recall in Table 2 shows that the HGBlock module has a lower precision but maintains a high recall while reducing metrics such as inference time, Params, and FLOPs. SPD-ADown improves the precision of the model. After module integration, the detection accuracy of the model in this paper met the requirements of tomato-picking robots. Furthermore, inference time was reduced by 1 ms with respect to the base model. By contrast, Params and FLOPs only rose by 3.8 M and 3.8 G. The model performance metrics in Table 4 are all values obtained after removing auxiliary branches.

### 4.2. Comparison Between Different Target Detection Networks

In order to test the actual detection effect of the improved algorithm, we compared and analyzed the improved algorithm with other classical target detection algorithms. The target detection algorithms for this comparison were SSD, Faster-RCNN, RetinaNet, YOLOv8 [33], and RT-DETR. Among them, the backbone network of SSD is MobileNetv2, the backbone network of Faster-RCNN and RetinaNet is ResNet101, and YOLOv8-l is used for YOLOv8 for comparison. The other models use the default structure. The experimental results are shown in Table 5.

Table 5 shows that the YOLOv9-all model in this paper has the highest detection accuracy with indicators mAP@0.5 and mAP@0.5:0.95 reaching 98% and 85.4%, respectively. The inference speed of this study was 14.7ms, which ranks second. The model’s Params and FLOPs values were also smaller than those of the YOLOv8 and RT-DETR models, which meet the needs of tomato-picking robots. Compared to YOLOv10 [34] and YOLOv11, the YOLOv9-all model has advantages in accuracy and detection speed but still falls short in Params and FLOPs. Nevertheless, our model is sufficient for use in practice.

**Figure 13:**
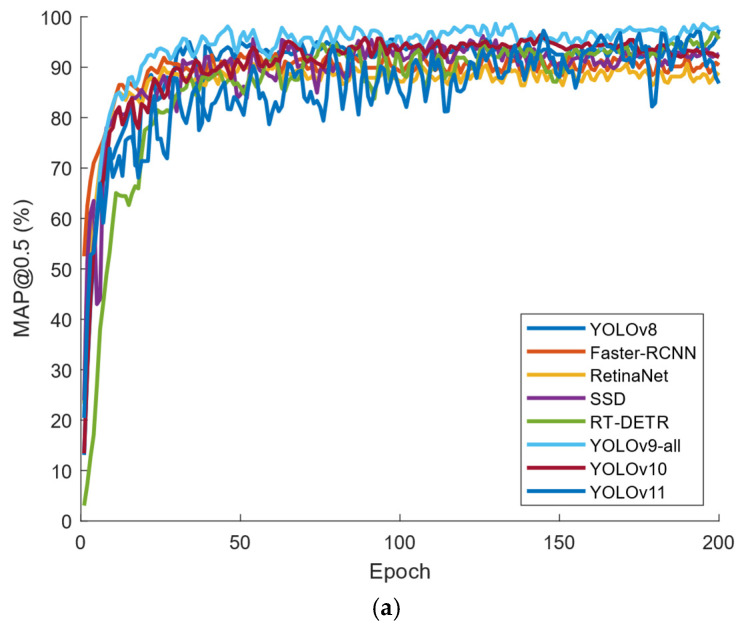

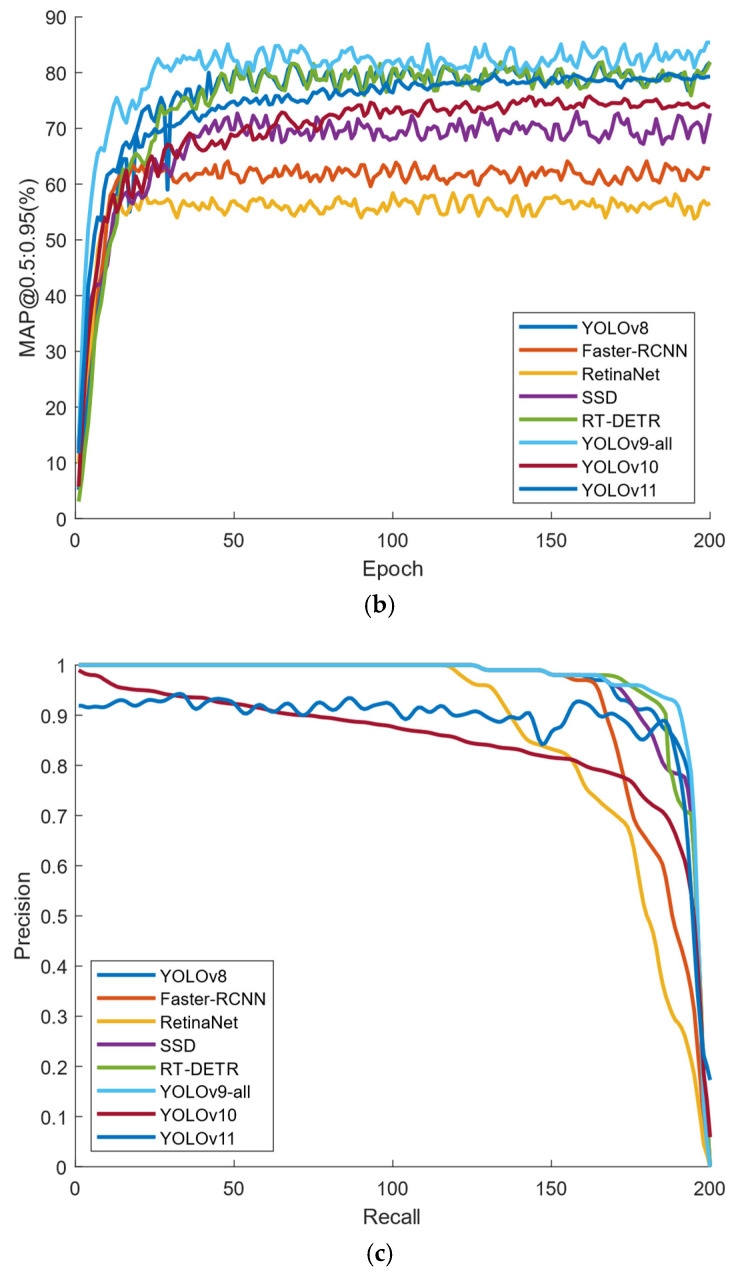
Comparison results of different models: (**a**) mAP@0.5; (**b**) mAP@0.5:0.95; (**c**) PR curve.

Furthermore, this paper shows the trend of mAP@0.5 and mAP@0.5:0.95 metrics on the test set during training. They are shown in Figure 13a,b, respectively. Figure 13c shows the transformation of PR curves for different models.

As seen in Figure 13a,b, the metrics of all models stabilize as the training proceeds, while the evaluation metrics of both mAP@0.5 and mAP@0.5:0.95 of the model proposed in this paper are significantly higher than the other models. The Precision–Recall Curve (PR curve) is an important tool for evaluating the performance of a target detection model, which reflects the change in accuracy relative to the recall of the model. Ideally, the PR curve should be as close as possible to the upper right corner of the axes, meaning that the model can maintain a high precision rate despite a high recall rate [35]. As seen in Figure 13c, the YOLOv9-all model in this paper clearly outperforms the other models on the PR curve, indicating its better overall performance in target detection.

**Figure 14:**
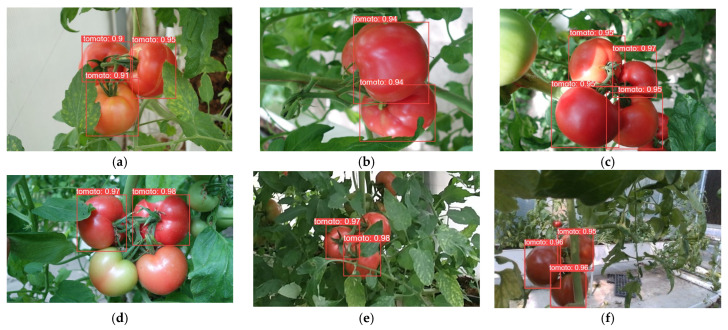
SSD algorithm performance: (**a**) micro-ripening tomato; (**b**) multi-fruit overlapping; (**c**) sunlight; (**d**) immature tomato; (**e**) leaf occlusion; (**f**) tomato stem occlusion.

**Figure 15:**
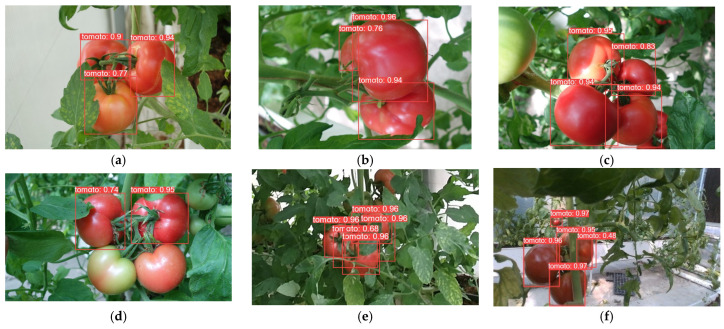
Faster-RCNN algorithm performance: (**a**) micro-ripening tomato; (**b**) multi-fruit overlapping; (**c**) sunlight; (**d**) immature tomato; (**e**) leaf occlusion; (**f**) tomato stem occlusion.

**Figure 16:**
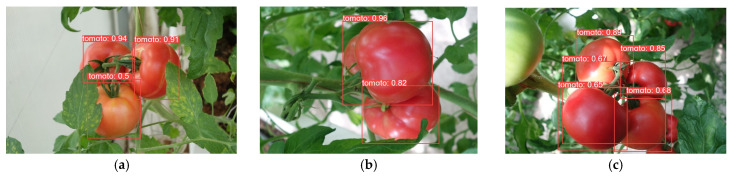

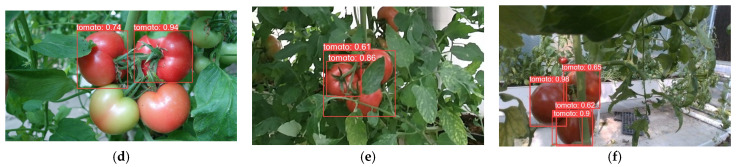
RetinaNet algorithm performance: (**a**) micro-ripening tomato; (**b**) multi-fruit overlapping; (**c**) sunlight; (**d**) immature tomato; (**e**) leaf occlusion; (**f**) tomato stem occlusion.

**Figure 17:**
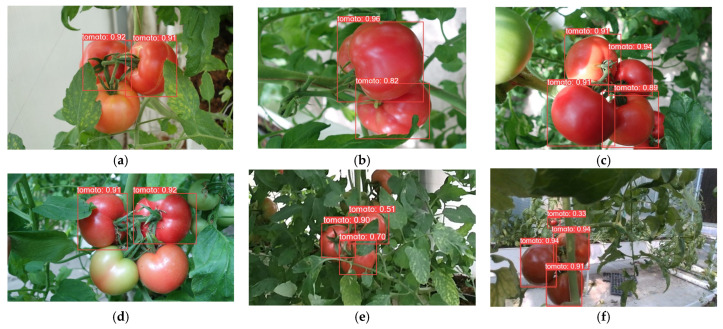
YOLOv8 algorithm performance: (**a**) micro-ripening tomato; (**b**) multi-fruit overlapping; (**c**) sunlight; (**d**) immature tomato; (**e**) leaf occlusion; (**f**) tomato stem occlusion.

**Figure 18:**
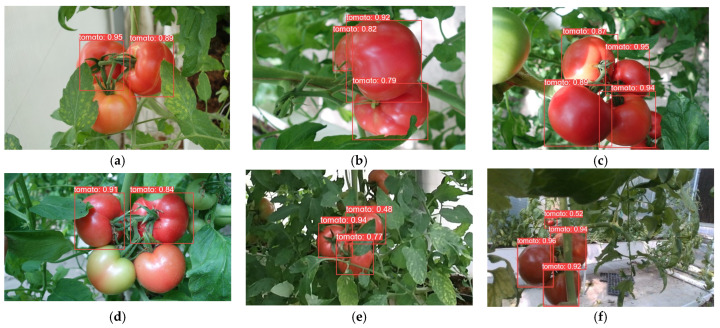
RT-DETR algorithm performance: (**a**) Micro-ripening tomato; (**b**) multi-fruit overlapping; (**c**) sunlight; (**d**) immature tomato; (**e**) leaf occlusion; (**f**) tomato stem occlusion.

**Figure 19:**
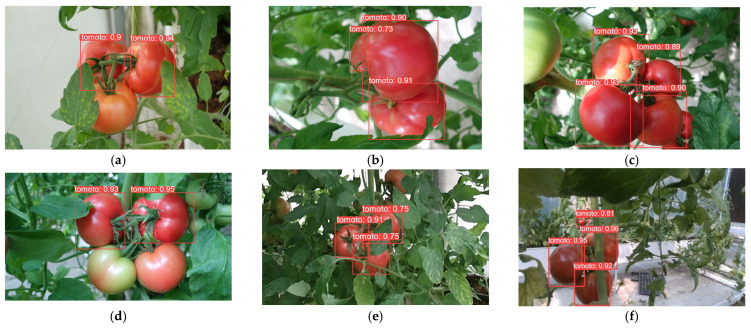
Algorithm performance in this article: (**a**) Micro-ripening tomato; (**b**) multi-fruit overlapping; (**c**) sunlight; (**d**) immature tomato; (**e**) leaf occlusion; (**f**) tomato stem occlusion.

In order to demonstrate the detection performance of different models, the results are visualized in this paper, as shown in Figure 14, Figure 15, Figure 16, Figure 17, Figure 18 and Figure 19 below. In this paper, six different scenarios were selected for comparison. These scenarios were categorized according to the following scenarios: mature tomatoes and slightly ripe tomatoes alternate, multiple fruits overlapping, toward the light, mature tomatoes and immature tomatoes alternate, leaf occlusion, and tomato stem occlusion.

As seen in Figure 19, the algorithms in this study have the best detection results. As seen in Figure 19a, SSD, Faster-RCNN, and RetinaNet incorrectly recognize slightly ripe tomatoes as ripe tomatoes. As seen in Figure 19f of YOLOv8 and the Figure 19f plot of RT-DETR, the confidence of detection frames for small targets is relatively low.

## 5. Discussion

This paper explores classical fruit recognition algorithms and examines the limitations of current fruit recognition approaches. The HGBlock module from the RT-DETR network was employed to replace the RepNCSPELAN4 feature extraction module in YOLOv9. This substitution enhanced computational efficiency and performed channel compression, reducing the algorithm’s spatial footprint while maintaining computational accuracy. As a result, the improved algorithm became more compatible with CPUs and other hardware. Inspired by other experiments, enhancing the ADown module with the SPD-conv module also improved the detection accuracy of small objects, addressing the limitations of YOLO’s detection capabilities. This development addresses the issue of low accuracy in detecting ripe tomatoes in complex environments while striving to maintain faster detection speed and higher accuracy, thus meeting tomato-picking robot needs.

The research results indicated that enhancing the feature extraction and downsampling modules of the YOLOv9 model is feasible. Comparative analysis showed that the improved model excels in computing speed and recall. Inferring Time was 14.7 ms, 20.7 ms faster than Faster-RCNN, 16 ms faster than RetinaNet, and 9.7 ms faster than YOLOv8. mAP@0.5 was 98%, 6.1% higher than Faster-RCNN, 9.6% higher than RetinaNet, and 2.4% higher than YOLOv8. The improved YOLOv9 had 6.1% and 1.0% higher detection accuracy and 21 ms and 13.7 ms faster inferring time than YOLOv10 and YOLOv11, respectively. Compared to the original model, the improved model also showed great improvement in inference speed and accuracy. The improved model became 1 ms faster in detection speed and mAP@0.5 improved by 5%. Enhancements to the feature extraction module reduced computational costs and hardware requirements while improving accuracy. The primary goal of this study was to achieve effective detection of ripe tomatoes, which is crucial for advancing tomato-picking technology. Compared to human eye recognition, the improved algorithm significantly reduced time and labor, contributing to automated tomato picking. More broadly, automated harvesting promotes increased agricultural productivity and optimization of the agricultural workforce structure, modernizing agriculture, and improving the quality of agricultural products [36]. Precise control of the picking process also protects the ecosystem and ecological balance from an environmental perspective. However, in the process of large-scale automation, the transition of the workforce may have some impact on socio-economic conditions and people’s well-being, as more labor positions will be needed. Until the broad economic prospects of fruit-picking robots can be demonstrated, obstacles involving farmers may arise prior to their introduction.

In practical applications, to improve fruit picking efficiency, it is necessary to continuously improve the speed and optimize the algorithm model. Despite substantial improvements, further optimization is required to reduce the algorithm’s FLOPs and refine its structure to enhance accuracy and recall. The improved model, which is slower than SSD in terms of detection speed, must be improved. Reducing the FLOPs will increase detection speed and save space to integrate more features into the robot. System robustness must also be improved to make the model suitable for more complex environments. In practical applications, uneven color distribution in tomatoes can impact detection accuracy, leading to erroneous results. Addressing this issue is necessary to enhance fruit recognition detection accuracy and promote automated tomato picking.

## 6. Conclusions

This study achieved improvements in detection speed and detection accuracy. The method was improved based on YOLOv9-C by replacing the feature extraction part RepNCSPELAN4 with the HGBlock module and the original convolutional layer with the SPD-conv module. The results show excellent detection results. mAP@0.5 reached 98.0%, a 5.0% increase over the original model, and far exceeded other target detection models. In the ablation experiment, the mAP@0.5 of this model was 0.2% higher than the model with only improved SPD-ADown and 0.5% higher than the model with only improved HGBlock. The detection speed reached 14.7 ms, which is 1 ms faster than the original model and 2.4 ms faster than the model with only improved HGBlock. This paper can improve detection speed and accuracy. The following conclusions can be drawn from this article:

(1) By introducing the HGBlock module instead of the original RepNCSPELAN4 module, the model reduces model complexity while maintaining detectability;
(2) This study improved the ADown module in the original YOLOv9 based on the SPD-conv module, which improved the accuracy of detecting small objects and low-resolution images as well as the robustness of the model;
(3) By comparing different algorithmic models, this model can reduce model complexity while ensuring detection performance, which improves detection speed and accuracy.

## Figures and Tables

**Table 1:** Specific parameters of Intel Realsense D435 camera.

Parameter Name	Parameter Value
Maximum RGB Image Resolution	1920 × 1080
Maximum Depth Image Resolution	1280 × 720
Ideal Detection Range	0.3 m~3 m
RGB Image Frame Rate	30 fps
Power Supply and Data Transmission Method	USB3.0

**Table 2:** Hyperparameter configuration table.

Parameter Item	Parameter Value
Number of Training Rounds	200
Number of Single Training Samples	4
Initial Learning Rate	0.01
Momentum	0.937
Optimizer	SGD

**Table 3:** Results of ablation experiment.

Model	SPD-ADown	HGBlock	mAP@0.5 (%)	mAP@0.5:0.95 (%)	Precision (%)	Recall (%)
Base	✕	✕	93.0	84.3	95.9	91.2
A	✓	✕	97.8	85.2	96.7	91.8
B	✕	✓	97.5	84.2	94.2	92.4
C	✓	✓	98.0	85.4	97.2	92.3

**Table 4:** Model evaluation.

Model	SPD-ADown	HGBlock	Inferring Time (ms)	Params (M)	FLOPs (G)
Base	✕	✕	15.7	25.3	102.1
A	✓	✕	17.1	31.6	118.7
B	✕	✓	13.0	22.9	90.4
C	✓	✓	14.7	29.1	105.9

**Table 5:** Comparison results of different models.

Target Detection Algorithm	mAP@0.5 (%)	mAP@0.5:0.95 (%)	Inferring Time (ms)	Params (M)	FLOPs (G)
SSD	94.3	73.3	10.9	3.1	0.7
Faster-RCNN	91.9	64.1	35.4	60.7	85.2
RetinaNet	88.4	58.4	30.7	55.3	74.1
YOLOv8	97.5	84.3	24.4	43.6	165.4
RT-DETR	95.6	81.9	29.1	61.8	191.4
YOLOv10	91.9	73.79	35.7	2.7	8.4
YOLOv11	97.0	79.3	28.4	2.6	6.6
YOLOv9-all	98.0	85.4	14.7	29.1	105.9

## Data Availability

The data used to support the results of this study have not been published because they were obtained by the authors and their institutions for a fee and involve the authors’ privacy.
